# The evolution of the macrophage-specific enhancer (Fms intronic regulatory element) within the CSF1R locus of vertebrates

**DOI:** 10.1038/s41598-017-15999-x

**Published:** 2017-12-07

**Authors:** David A. Hume, Evi Wollscheid-Lengeling, Rocio Rojo, Clare Pridans

**Affiliations:** 1 Mater Research-University of Queensland, Translational Research Institute, Woolloongabba, Brisbane, Australia; 2 The Roslin Institute, University of Edinburgh, Easter Bush, Midlothian, UK

## Abstract

The *Csf1r* locus encodes the receptor for macrophage colony-stimulating factor, which controls the proliferation, differentiation and survival of macrophages. The 300 bp Fms intronic regulatory element (FIRE), within the second intron of *Csf1r*, is necessary and sufficient to direct macrophage-specific transcription. We have analysed the conservation and divergence of the FIRE DNA sequence in vertebrates. FIRE is present in the same location in the *Csf1r* locus in reptile, avian and mammalian genomes. Nearest neighbor analysis based upon this element alone largely recapitulates phylogenies inferred from much larger genomic sequence datasets. One core element, containing binding sites for AP1 family and the macrophage-specific transcription factor, PU.1, is conserved from lizards to humans. Around this element, the FIRE sequence is conserved within clades with the most conserved elements containing motifs for known myeloid-expressed transcription factors. Conversely, there is little alignment between clades outside the AP1/PU.1 element. The analysis favours a hybrid between “enhanceosome” and “smorgasbord” models of enhancer function, in which elements cooperate to bind components of the available transcription factor milieu.

## Introduction

Transcriptional regulation in eukaryotes involves a complex interaction between distal regulatory elements (enhancers) and proximal promoters. Most eukaryotic genes are influenced by multiple enhancers which may display a degree of redundancy and which appear to evolve more rapidly than promoters^1,2^. Despite their rapid evolution, many enhancers are sufficiently conserved to permit their identification based upon sequence conservation (phylogenetic footprinting). Comparative analysis with other mammalian genomes indicated that 3–8% of the human genome has been subject to purifying selection, most of which is not protein-coding and inferred to be regulatory^3,4^. Most enhancers contain binding sites for multiple transcription factors, and despite overall conservation, individual binding sites may be gained and lost through alterations in DNA sequence with consequential changes in gene regulation. Early comparative analysis of functional elements identified in human promoters indicated around 30–40% are lost in mouse^5^. One extreme example is the absolute divergence in glucorticoid-inducible gene expression between humans and mice as a consequence of the gain and loss of glucocorticoid-receptor binding to distal enhancers^6^. Other examples have been reviewed by Villar *et al*.^3^.

There are two prevailing models for the function of the individual binding motifs, and the factors that bind them, within a complex enhancer. In some enhancers, the cooperative binding of multiple transcription factors in a precise array is required for activity, and each element is non-redundant. The complex of bound transcription factors has been referred to as an enhanceosome. In the alternative “billboard” model, each transcription factor binds to the enhancer, and interacts with the promoter, to some extent independently to regulate transcription^1^. Such a model is favoured by the increasing recognition of the probabilistic basis of transcriptional regulation at the single cell level^7–9^. A “billboard” type enhancer may have a conserved function despite a relative lack of alignable sequence conservation^3^.

The differentiation of vertebrate macrophages is controlled by signals from the macrophage colony-stimulating factor (CSF1) receptor, CSF1R (also known as the c-fms protooncogene) which has two ligands, CSF1 and interleukin 34 (IL34). This function of CSF1R and the two ligands in macrophage differentiation is conserved from bony fish and birds through to humans^10,11^. The transcription regulation of *Csf1r* in mouse and human has been studied extensively^12^. The second intron, downstream of the first coding exon, contains a conserved 300 bp regulatory element, the Fms intronic regulatory element, or FIRE. FIRE is an unusual enhancer in that the activity is position and orientation dependent, and is associated with the generation of an antisense transcript^13^. The presence of FIRE is essential to the activity of a *Csf1r* transgenic reporter gene in mice^14^. A lentiviral vector containing FIRE and the *Csf1r* promoter was able to direct macrophage-restricted reporter gene expression in mouse, rat, human, pig, cow, sheep, and even chicken macrophages *in vitro* ^15^ and in transgenic sheep^16^. So, FIRE is both necessary and sufficient to direct macrophage-specific transcription from the *Csf1r* locus. The chicken *Csf1r* locus contains a regulatory sequence in the same relative location as FIRE that is conserved between bird species and can direct expression of reporter genes to the macrophage lineage in transgenic chick^17^.

The mouse FIRE sequence contains binding sites for numerous macrophage-expressed transcription factors, including PU.1, KLF4, RUNX1, CEBP and AP1 family members^12^. The rapid decline in DNA sequencing costs has increased the availability of genomic DNA sequences from many more distantly-related species which offers the opportunity to analyse the way in which FIRE has evolved across species. Here we present an analysis of the conservation and divergence of vertebrate FIRE sequences.

## Methods

All analysis was carried out using the MacVector^TM^ (Apex, NC, USA) programme. Mammalian, avian and reptilian FIRE sequences were individually extracted from completed genomes and whole genome sequencing available in NCBI (https://www.ncbi.nlm.nih.gov) using “BLAST Genomes”, with mouse or human (for mammals), chicken or zebra finch (for birds) and alligator or anole lizard (for reptiles) as the query. The most conserved sequence that was also specifically associated with the *Csf1r* locus was aligned with the query and trimmed accordingly. For the snakes, there was no initial BLAST hit using available query sequences on any snake genome draft assembly in NCBI. We therefore extracted the second intron of the annotated *Csf1r* locus from available snake genomic sequences, and using Pustell, identified a 300 bp conserved region in the same relative location as FIRE in birds that contained the core elements described below. That sequence was then used in BLAST to identify similar sequences in other snake genomes. All of the sequences analysed are provided in the alignments in Supplementary Figures.

The representation of available FIRE sequences from non-placental mammals was relatively low compared to placental mammals. Only platypus, echidna (monotremes), Tasmanian devil, koala and opossum were available. A partial FIRE sequence was detected in the Tamar Wallaby genome, interrupted by Ns. To extend the available marsupial sequences, we obtained DNA from 5 species (Long-nosed Potoroo (*Potorus tridactylus*), Southern brown bandicoot (*Isoodon obesulus*), Western grey kangaroo (*Macropus fuliginosus*), Western quoll (*Dasyurus viverrinus)* and Fat-tailed dunnart, (*Sminthopsis crassicaudata*)^18^.

Based upon the known conserved flanking sequences of marsupial FIRE sequences, we designed PCR primers (FIREMarsupialFWD: 5′AAGCAGAAGTGAGAGAATATGTGTGGG and FIREMarsupialREV: 5′ GTTTTCTTTTAAGGAACTTTTCTTG) to amplify the sequence. PCR cycles were performed as follows: an initial denaturing step 95 °C for 3 min, followed by 30 cycles of 95 °C for 30 s, 55 °C for 30 s, 72 °C for 45 s, and an elongation step 72 °C for 3 min. PCR products obtained from these species were purified using the QIAquick PCR Purification Kit following the manufacturer’s protocol and sequenced by Sanger sequencing at the Institute for Genetics and Molecular Medicine (IGMM), University of Edinburgh. Genebank IDs are *Isoodon* MG014607; *Macropus*, MG014608; *Dasyurus* MG014609; *Potorus;* MG014610; *Sminthopsis*, MG014611.

To generate phylogenetic trees, and consensus sequences, the sequences from each clade were trimmed to a common length, and aligned using ClustalW. Neighbour joining trees were generated using MacVector. The distance measures shown in the resulting phylogenetic trees (uncorrected “P” values) represent the proportion (*p*) of nucleotide sites at which two sequences being compared are different. It is obtained by dividing the number of nucleotide differences by the total number of nucleotides compared. It does not make any correction for multiple substitutions at the same site, substitution rate biases (for example, differences in transition or transversion rates) or differences in evolutionary rates among sites. The set of candidate regulatory motifs with the consensus sequence from each clade was identified by scanning the sequence using the Jaspar motif database (http://jaspar.genereg.net), allowing for >85% match to the position weight matrices. As noted by Sandelin & Wasserman^19^, the binding sites recognised by multiple members of transcription factor families can be grouped and motif analysis alone cannot distinguish which family member is likely to bind. Accordingly, we grouped the putative transcription factors identified with Jaspar by families and highlighted those with known expression in macrophages^12^.

## Results and Discussion

**Figure 1:**
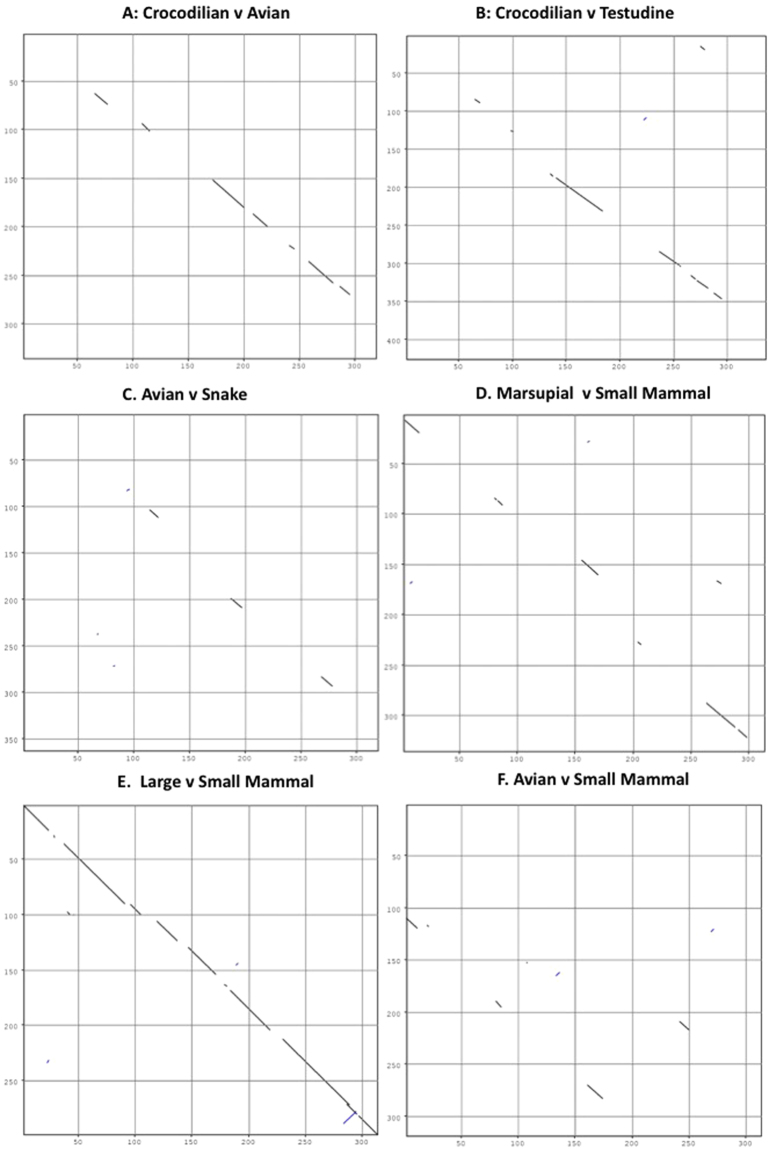
Alignment of consensus FIRE sequences from various clades. Dot matrix alignment was performed using the Pustell algorithm in MacVector, with a window size of 15 and minimal identity of 70%. Consensus sequences are the same as in Fig. 5, derived from Figures S1–S5. In each case the first named clade is on the Y axis.

Figureentary Figures S1–5 show the alignment of the reptile, avian, small placental mammal (mainly rodents) large placental mammal and marsupial/monotreme FIRE sequences. The separation of the small and large animals was based in part upon previous analysis that revealed around 90% conservation of the FIRE sequence between mouse and human, and evidence from other genomic analysis favouring a primate/artiodactyl split, with rodents as an outgroup^20^. Whereas the avian and the small and large animal mammal sequences showed very substantial within-clade alignment and homology, the monotremes (platypus and echidna) were very divergent from the marsupials and the reptile sequences were even more divergent amongst the major groups (lizards, snakes, crocodilians and turtles). Indeed, the three available lizard sequences (the anole lizard, Japanese gecko and bearded dragon) were also very divergent from each other and there was only a weak consensus. We extracted the consensus sequences from mammals, birds, snakes, crocodiles and turtles from the ClustalW alignments. Figure 1 shows a Pustell matrix alignment of a number of these consensus sequences. As shown in Fig. 1A and B, the crocodilian and turtle FIRE sequences partly aligned with avian sequences but the snakes were much more divergent (Fig. 1C). Amongst the mammals, marsupials were clearly divergent (Fig. 1D), whereas the small and large placental mammals, were closely-related with only small areas of disalignment (Fig. 1E). Finally, alignment of birds and mammals revealed that there was an incomplete overlap, with avian-specific and mammal-specific regions outside the core element (Fig. 1F).

**Figure 2:**
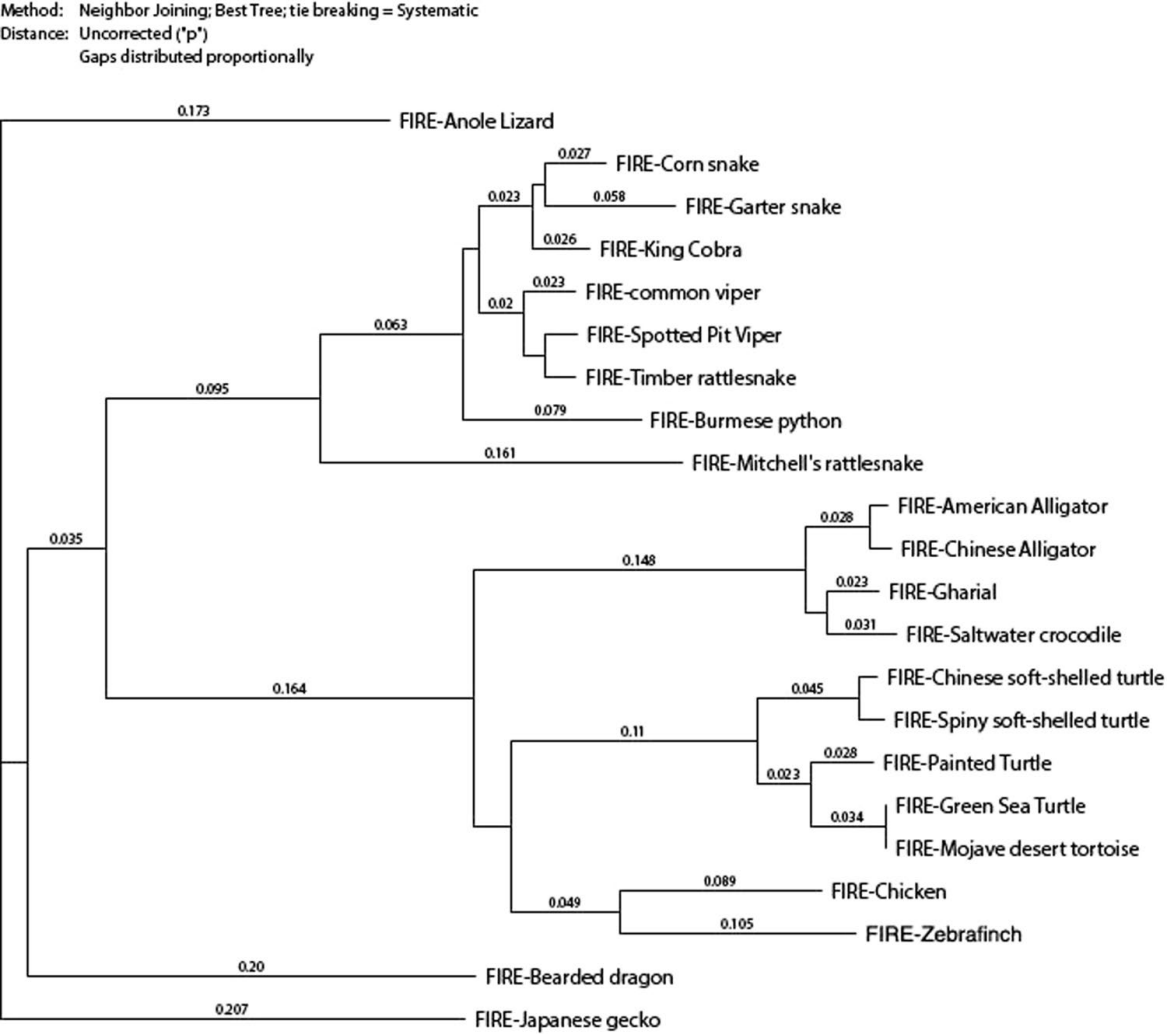
Neighbour-joining tree of the reptile clade (with chicken and zebrafinch). Neighbour joining tree was generated based upon the ClustalW alignments shown in Figure S2, as described in Methods. The branches show uncorrected P values (*p*), the proportion of nucleotide sites at which two sequences being compared are different.

**Figure 3:**
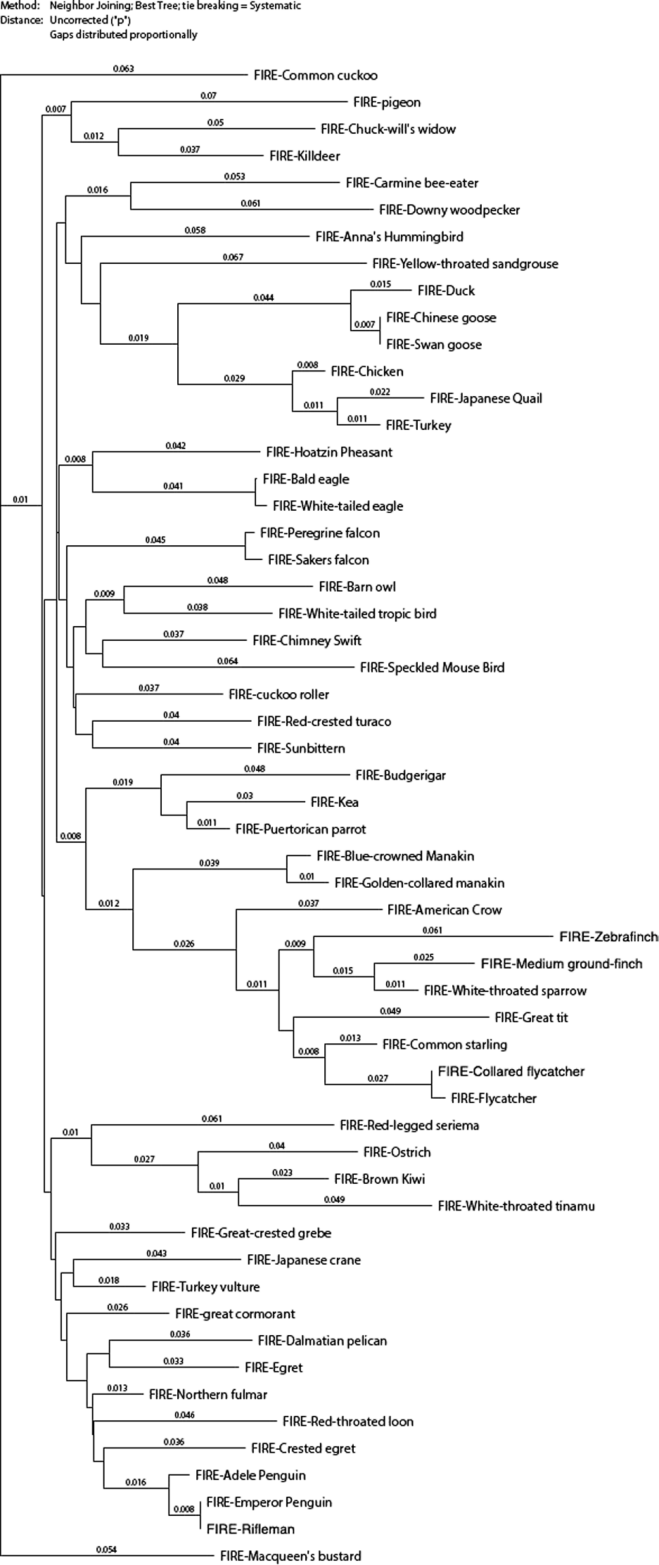
Neighbour-joining tree of the avian clade. Neighbour joining tree was generated based upon the ClustalW alignments shown in Figure S1, as described in Methods. The branches show uncorrected P values (*p*), the proportion of nucleotide sites at which two sequences being compared are different.

**Figure 4:**
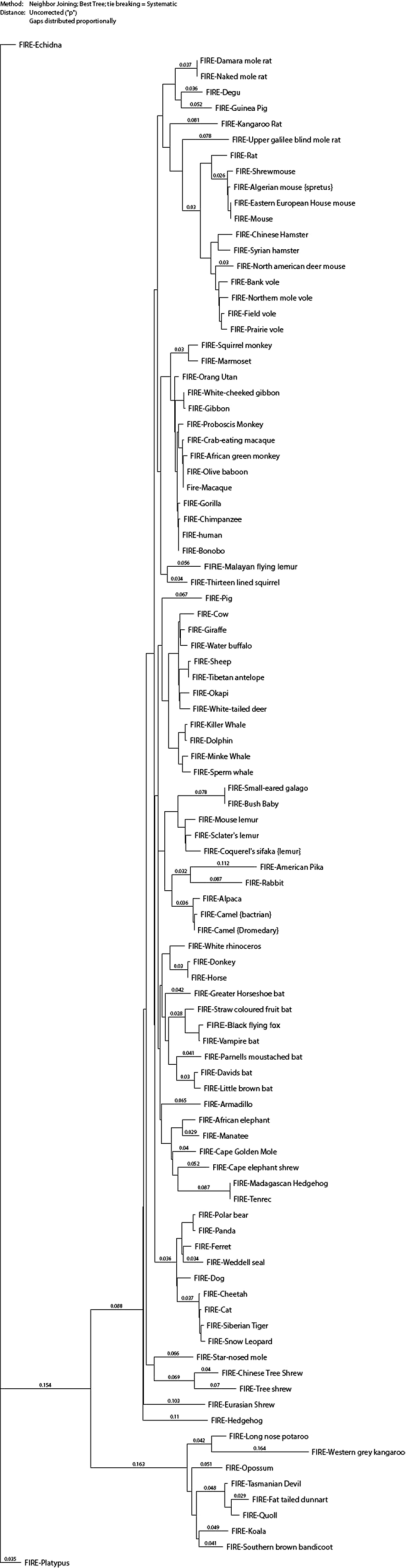
Neighbour-joining tree of the mammalian clade (including marsupials and monotremes). Neighbour joining tree was generated based upon combination of the ClustalW alignments shown in Figures S3–S5, as described in Methods. The branches show uncorrected P values (*p*), the proportion of nucleotide sites at which two sequences being compared are different.

Based upon the ClustalW alignments we generated neighbor-joining trees for each Clade based solely upon the FIRE sequences. Although there was a clear link between the reptile and bird sequences, for ease of visualization, the reptiles are shown separately, with two bird sequences (chicken and zebrafinch) included for comparison (Fig. 2). The avian tree is shown in Fig. 3 and the mammalian in Fig. 4. There are obvious parallels with more sophisticated phylogenetic analysis based upon maximum parsimony and much larger datasets. For example, the simple nearest neighbor groupings based upon FIRE almost perfectly recapitulate the broad divisions of bird species based upon analysis of whole genomes^21^ and support the close relationship between the crocodilians, testudines (turtles and tortoises) and birds (Fig. 2). In mammals, the monotremes form the base of the phylogenetic tree, with marsupials (including the opossum) making a clear branch. As inferred from the analysis of genomic retrotransposon insertions^18^, the Tasmanian devil, quoll and fat-tailed dunnart (Dasyuromorphia) were clearly associated in one branch. The groupings of the placental mammals are largely consistent with conclusions based upon 447 nuclear genes in 37 species^22^. In this respect, FIRE is representative of the class of conserved non-exonic elements (CNEE) that have been analysed as promising phylogenetic markers in birds, and indeed the tree in Fig. 3 largely matches the avian phylogenetic relationships derived from >3800 CNEE in a smaller set of diverse bird species^23^. As suggested by these authors, FIRE, as a typical CNEE, provides a positional framework for phylogenetic analysis, anchored on blocks of substantially conserved sequences (the transcription factor binding sites), between which base substitutions/inclusions/deletions are not constrained and their drift can provide indications of evolutionary relationships.

The conserved sequence blocks are clearly constrained by the binding affinity of the transcription factors that bind them. The precise regulation of mouse and human FIRE by myeloid-specific transcription factors has been reviewed recently^12^. Combinations of ChIP-seq and *in vivo* footprinting indicate that the conserved sequences with FIRE in both species are occupied by transcription factors including PU.1, AP1, CEBPA/B, STAT1, IRF8, KLF4 and RUNX1^12^.

**Figure 5:**
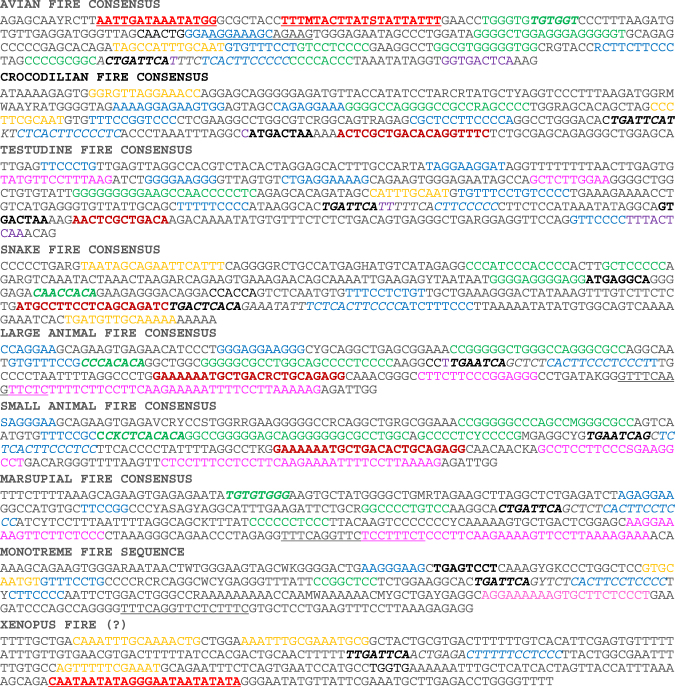
Candidate transcription factor binding motifs within the FIRE sequences of different clades. The consensus sequences of FIRE from each clade were derived from Figures S1 to S5. Each sequence was searched for motifs at a stringency of >0.85 using Jaspar, and related motifs were grouped into families. Candidate motifs within each FIRE sequence are highlighted as follows:       **XXX AP1 (Fos-Jun family)**  XXX IRFB The cosnerved AP1/PU.1 motif is italicized in each sequence.

**Figure 6:**
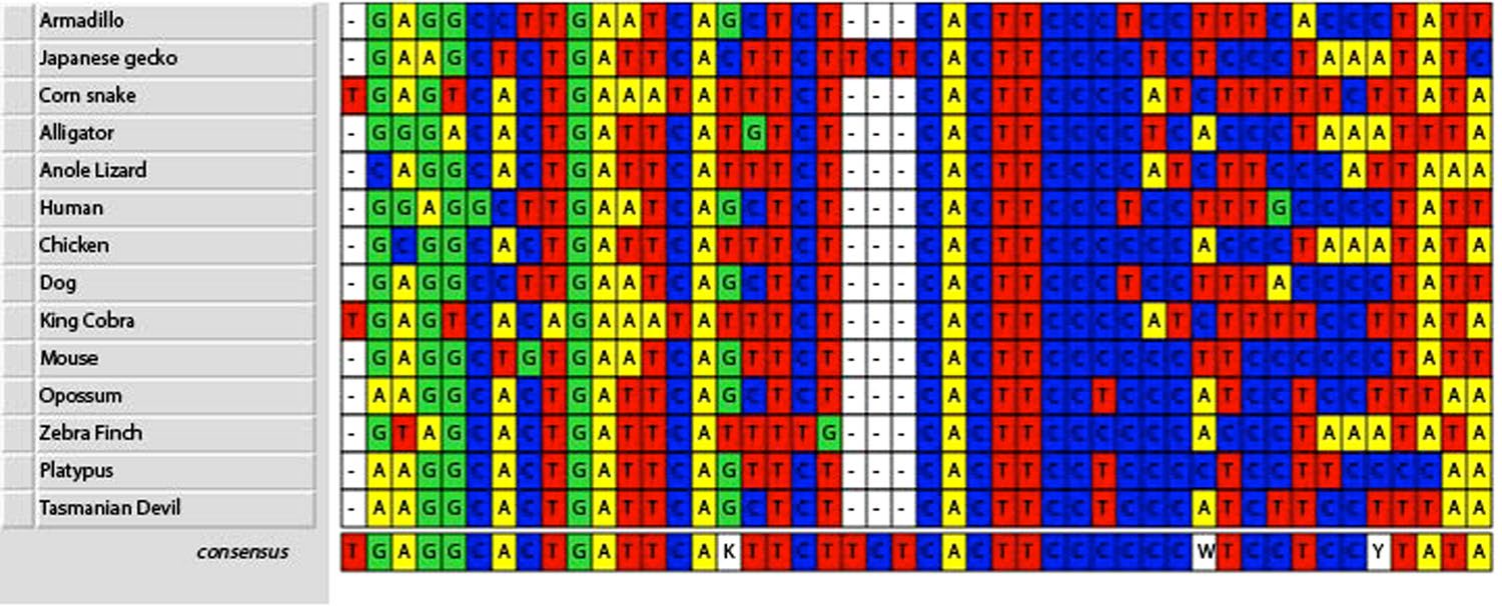
Clustal W alignment of the conserved AP1/PU.1 element within FIRE. Clustal W alignment was performed using the MacVector programme as described in Methods.

In Fig. 5, we summarise the candidate transcription factors that bind to consensus sequences of each of the animal families, derived from the alignments in Figures S1–S5. The only element of the FIRE sequence found in all of the species is shown in Fig. 6, with representatives from each of the major groups. The core motif, CACTTCCYY (RRGGAAGTG), matches the high affinity binding site for the Ets family macrophage-specific transcription factor, PU.1 (encoded by the *SPI1* gene) determined by ChIP-seq analysis of human monocytes and monocyte-derived macrophages^24^ and mouse macrophages^25^. The FIRE PU.1 site is occupied in mouse macrophage progenitor cells^26^ and PU.1 is essential for *Csf1r* expression in cytokine-dependent granulocyte-macrophage progenitors^27^. Several quite divergent species, including mouse and platypus shown in Fig. 6, have additional repeated purine-rich motifs within FIRE that may bind PU.1 or another Ets family transcription factor. The macrophage-specific promoters of mammalian *Csf1r* genes also vary in the number of PU.1 binding sites, with evidence of cooperative activity of different Ets family members^28^. The ChIP-seq analysis of PU.1 binding in mouse and human also revealed strong enrichment for AP1 (Fos/Jun) consensus motifs in the immediate vicinity of PU.1 binding sites^24,25^. The core of the FIRE element also contains a conserved consensus AP1 site that is essential for the enhancer and promoter activity of mouse FIRE *in vitro* ^13^. The precise apposition and orientation suggests that there might be cooperative binding to this motif. Combinatorial interactions between JUN and PU.1 have been noted in the regulation of other macrophage-specific enhancers^29–31^ and there have been multiple reports of direct physical interaction between PU.1 and JUN family members (Reviewed in ref.^32^). Comparative analysis of variations in PU.1 binding amongst mouse strains indicated that strain-specific PU.1 binding often involved variation in adjacent AP1 motifs^25^. One surprising feature of the AP1 element (TGAWTCA) is that the central base (A/T) is consistent from lizards to humans, and the motif is distinct from the classical AP1 consensus (TGASTCA). The one exception is in snakes, where the AP1 element is the consensus, TGAGTCA, and it is displaced by around 6–7 bp from the PU.1 element. We speculate that the variant AP1 element might either bind AP1 complexes with relatively low affinity (therefore requiring cooperativity with PU.1), or might bind specific members of the Fos/Jun/ATF family selectively, promoting effective interaction. This core AP1/Ets element resembles the distal regulatory element that has been characterised in detail in the mouse urokinase plasminogen activator (*Plau*) gene that responds to tyrosine kinase-Ras-Raf-Map kinase signals^33,34^. In the *Plau* enhancer the AP1 site (TGAGGTCA) is also distinct from the consensus. Growth factor signals in progenitor cells acting on the weak AP1 element within FIRE could form part of the initial chromatin remodelling allowing the binding of PU.1 and other factors. The CSF1/CSF1R regulatory mechanism and macrophage-restricted expression of *Csf1r* is conserved in *Xenopus* ^35^. Although we could not confirm function or conservation of a FIRE-like element in amphibia, having access to only the xenopus sequence, a BLAST search of the *Xenopus Csf1r* locus revealed a candidate AP1/PU.1 motif in the same relative location as FIRE in other species, suggesting that this basic mechanism may have arisen very early in evolution. The sequence of this region is shown in Fig. 5. In bony fish, the *Csf1r* locus is duplicated; one copy, *Csf1ra*, appears to be expressed in macrophages and mutations compromise early macrophage development in zebrafish^36^. A macrophage-expressed *Csf1r* cDNA has been isolated in several other fish species^37^. However, we have not detected any aligned regions, nor any AP1 motifs within the introns of available fish *Csf1ra* genomic sequences, nor any sequences matching the conserved PU.1/AP1 element anywhere in the genomes of cartilaginous fish. Hence, it appears that this core element arose in the land vertebrates.

The various conserved elements surrounding the PU.1/AP1 site in FIRE are annotated in Fig. 5. Each of them conforms to the consensus binding sites for known macrophage-expressed transcription factors, including additional PU.1/Ets sites (but likely lower affinity). However, consistent with the lack of extended sequence alignments in Fig. 1, the FIRE sequences from the different clades contain idiosyncratic sets of candidate macrophage-specific transcription factor binding sites in distinct positions relative to each other. Even within clades, some binding sites are probably gained or lost. There are two binding sites for Runx1 in mouse FIRE. The higher affinity Runx1 binding site that was characterised in detail^38^ is conserved only in murids. In other rodent species, this element has base substitutions that would most likely abolish Runx1 binding (Figure S3). In other animals, including humans, it is completely absent and only the second, lower affinity site is retained (Figure S4).

The transcriptional regulation of *Csf1r* has assumed clinical importance because of the identification of dominant mutations in the gene associated with a human autosomal dominant neurodegenerative disease^39^. In principle, the penetrance/expressivity of such mutations could depend in part on the level of expression of the wild-type allele. Table S1 shows the alignment of FIRE across higher primate species. FIRE is 100% identical in human and bonobo, and differs only by 1 bp in chimpanzee and gorilla. The Table also highlights bases that are variant in dbSNP for humans on NCBI. All are GC, or CG transversions within GC-rich elements, and none has a significant minor allele frequency. Hence, variation in FIRE is unlikely to contribute to the pathology of human neurodegenerative disease.

The pattern of motif shuffling that we observe (Fig. 5) amongst clades in the evolution of the FIRE DNA sequence suggests a hybrid between the enhanceosome and “billboard” models of transcriptional regulation. The archetypal enhanceosome is the 55 bp element of the *IFNB1* locus, which binds at least 7 different inducible transcription factors with a precise topology^40^. The *IFNB1* enhanceosome is almost perfectly conserved amongst mammals. The entire 300 bp FIRE sequence is highly-conserved in mammals, almost 90% conserved between mouse and human and most of the putative binding sites shown in Fig. 5 are probably occupied by transcription factors in mouse macrophages^12^. The ancestral versions of FIRE in other clades may have arisen by the aggregation of regulatory sites around the core functional PU.1/AP1 motif to produce a functional enhancer that is able to sample the available “smorgasbord” of myeloid transcriptional factors. We have confirmed in RNA-seq analysis that chicken bone marrow-derived macrophages express the same sets of transcription factors as mouse (Ms in preparation). The precise location and indeed the identity of the transcription factor motifs varies between clades. As we have noted previously, the same motifs exist in the *Csf1r* promoter, and point mutations in adjacent functional Runx1 and CEBP elements identified in the human promoter produce a loss of binding in the mouse promoter^41,42^. In mammals, there is an extensive conserved STAT/IRF8 motif within FIRE, and binding of STAT1 and IRF8 has been confirmed in ChIP-seq analysis of mouse macrophages^12^. The extended STAT/IRF motifs present in mammalian FIRE are not obvious in avian FIRE (although there is a novel candidate IRF8 motif elsewhere in the element), nor are the AT-rich (FOX family) sequences in avian FIRE present in mammals, but the mouse FIRE sequence is functional as an enhancer in chicken macrophages^15^. The conservation of sequence within clades suggest that the gain and loss of motifs has been constrained within each clade on the basis that the loss of any one site produces a significant reduction in transcriptional activity, as observed in the *IFNB1* enhanceosome. That is certainly the case for the core AP1 element, and for RUNX1 binding sites that we have assayed directly^13,38^. By analogy, the loss of a single transcription factor binding site in the conserved long range enhancer of the *Shh1* locus is associated with the loss of limb development in snakes^43^.

The evolutionary conservation of core elements of FIRE suggests that there would be a phenotype associated with a loss of function. We are currently analysing the knockout of this element in mice, which does indeed impact on *Csf1r* transcription (Rojo *et al*. Manuscript in preparation). Interestingly, and despite the fact that CSF1 signaling down-regulates *Csf1r* mRNA by acting on the anti-sense promoter activity of FIRE^13,42^ a heterozygous knockout of *Csf1r* in mice^44^ and rats (CP, DAH, Manuscript in preparation) is not dosage-compensated and produces a 50% reduction in *Csf1r* mRNA. Accordingly, a heterozygous loss of function of FIRE could produce an impact on macrophage biology that might produce a selective advantage or disadvantage.

In summary, the FIRE sequence alone can be amplified using generic primers, and provides approximate indications of phylogenetic relationships amongst species. The core AP1/PU.1 sequence arose early in vertebrate evolution, and in different clades this element has associated with a cohort of binding sites that sample the myeloid transcription factor landscape.

## Electronic supplementary material

Supplementary Figures and Tables
