# Supplementary material for: The evolution of the macrophage-specific enhancer (Fms intronic regulatory element) within the CSF1R locus of vertebrates
Source: Sci Rep. 2017 Dec 7;7:17115. doi: 10.1038/s41598-017-15999-x (PMC5719456; doi:10.1038/s41598-017-15999-x)

Supplementary Figures

The evolution of the macrophage-specific enhancer (Fms intronic regulatory element) within the CSF1R locus of vertebrates

David A. Hume[1,2], Evi Wollscheid-Lengeling[2], Rocio Rojo[2] and Clare Pridans[2]

1. Mater Research-University of Queensland, Translational Research Institute, Woolloongabba, Brisbane, Australia
2. The Roslin Institute, University of Edinburgh, Easter Bush, Midlothian, UK

Figure S1. ClustalW alignment of the avian FIRE sequences

Alignment was carried out using MacVector, and consensus sequence is shown at bottom. Conserved AP1/PU.1 element is at 255-275 in the zebrafinch sequence.

Figure S2. ClustalW alignment of the reptile FIRE sequences

Alignment was carried out using MacVector, and consensus sequence is shown at bottom. Conserved AP1/PU.1 element is at 255-275 in the zebrafinch sequence.

Figure S3. ClustalW alignment of the small mammal FIRE sequences

Alignment was carried out using MacVector, and consensus sequence is shown at bottom. Conserved AP1/PU.1 element is at 130-150 in the mouse sequence.

Figure S4. ClustalW alignment of the large mammal FIRE sequences

Alignment was carried out using MacVector, and consensus sequence is shown at bottom. Conserved AP1/PU.1 element is at 130-150 in the human sequence.

Figure S5. ClustalW alignment of the marsupial and monotreme FIRE sequences

Alignment was carried out using MacVector, and consensus sequence is shown at bottom. Conserved AP1/PU.1 element is at 152-172.

TABLE S1. Alignment of primate FIRE sequences

```
FIRE-human       GGAAGCAGAAGTGAGAACATCCCTGTAGAAGGGCCACAGGCTGAGCGGAAACCGGGGGCTGAGCCTGACGCCAACAATGT
FIRE-Orang Utan GGAAGCAGAAGTGAGAACATCCCTGTAGAAGGGCCACAGGCTGAGCGGAAACCGGGGGCTGAGCATGACGCCAACAATGT
FIRE-Bonobo      GGAAGCAGAAGTGAGAACATCCCTGTAGAAGGGCCACAGGCTGAGCGGAAACCGGGGGCTGAGCCTGACGCCAACAATGT
FIRE-Chimpanzee GGAAGCAGAAGTGAGAACATCCCTGTAGAAGGGCCACAGGCTGAGCGGAAACCGGGGGCTGAGCCTGACGCCAACAATGT
FIRE-Gorilla     GGAAGCAGAAGTGAGAACATCCCTGTAGAAGGGCCACAGGCTGAGCGGAAACCAGGGGCTGAGCCTGACGCCAACAATGT
                ***************************************************** ********** ***************

FIRE-human       GTTTCCGCCCACACAGGCTGGGGGGCGCCTGGCAGCCCCTCGGAGGCTTGAATCAGCTCTCACTTCCCTCCTTTGCCCCT
FIRE-Orang Utan GTTTCCGCCCACACAGGCTGGGGGGCGCCTGGCAGCCCCTCGGAGGCTTGAATCAGCTCTCACTTCCCTCCTCTGCCCCT
FIRE-Bonobo      GTTTCCGCCCACACAGGCTGGGGGGCGCCTGGCAGCCCCTCGGAGGCTTGAATCAGCTCTCACTTCCCTCCTTTGCCCCT
FIRE-Chimpanzee GTTTCCGCCCACACAGGCTGGGGGGCGCCTGGCAGCCCCTCGGAGGCTTGAATCAGCTCTCACTTCCCTCCTTTGCCCCT
FIRE-Gorilla     GTTTCCGCCCACACAGGCTGGGGGGCGCCTGGCAGCCCCTCGGAGGCTTGAATCAGCTCTCACTTCCCTCCTTTGCCCCT
                ************************************************************************ *******

FIRE-human       ATTTTAGGCCCTGGAAAAATGCTGACGCTGCAGAGGCAACGGGCCTTCTTCCCGGACAGCCTGATAGGGGTTTCAAGTTC
FIRE-Orang Utan ATTTTAGGCCCTGGAAAAATGCTGACGCTGCAGAGGCAACGGGCCTTCTTCCCGGACGGCCTGATAGGGGTTTCAAGTTC
FIRE-Bonobo      ATTTTAGGCCCTGGAAAAATGCTGACGCTGCAGAGGCAACGGGCCTTCTTCCCGGACAGCCTGATAGGGGTTTCAAGTTC
FIRE-Chimpanzee ATTTTAGGCCCTGGAAAAATGCTGACGCTGCAGAGGCAACAGGCCTTCTTCCCGGACAGCCTGATAGGGGTTTCAAGTTC
FIRE-Gorilla     ATTTTAGGCCCTGGAAAAATGCTGACGCTGCAGAGGCAACGGGCCTTCTTCCCGGACAGCCTGATAGGGGTTTCAAGTTC
                **************************************** **************** **********************

FIRE-human       TCTTTTCTCCTTCAAGAAAATTTTCCTTAAAAGAGATTG
FIRE-Orang Utan TCTTTTCTCCTTCAAGAAAATTTTCCTTAAAAGAGATTG
FIRE-Bonobo      TCTTTTCTCCTTCAAGAAAATTTTCCTTAAAAGAGATTG
FIRE-Chimpanzee TCTTTTCTCCTTCAAGAAAATTTTCCTTAAAAGAGATTG
FIRE-Gorilla     TCTTTTCTCCTTCAAGAAAATTTTCCTTAAAAGAGATTG
                ***************************************
```

Sequences were aligned using ClustalW. The red highlight on the human sequence locates the position of variants from this reference sequence on ENSEMBL variation tables, which includes data from the thousand genomes. None of the variants has a significant population frequency

Figure S1

Formatted Alignments

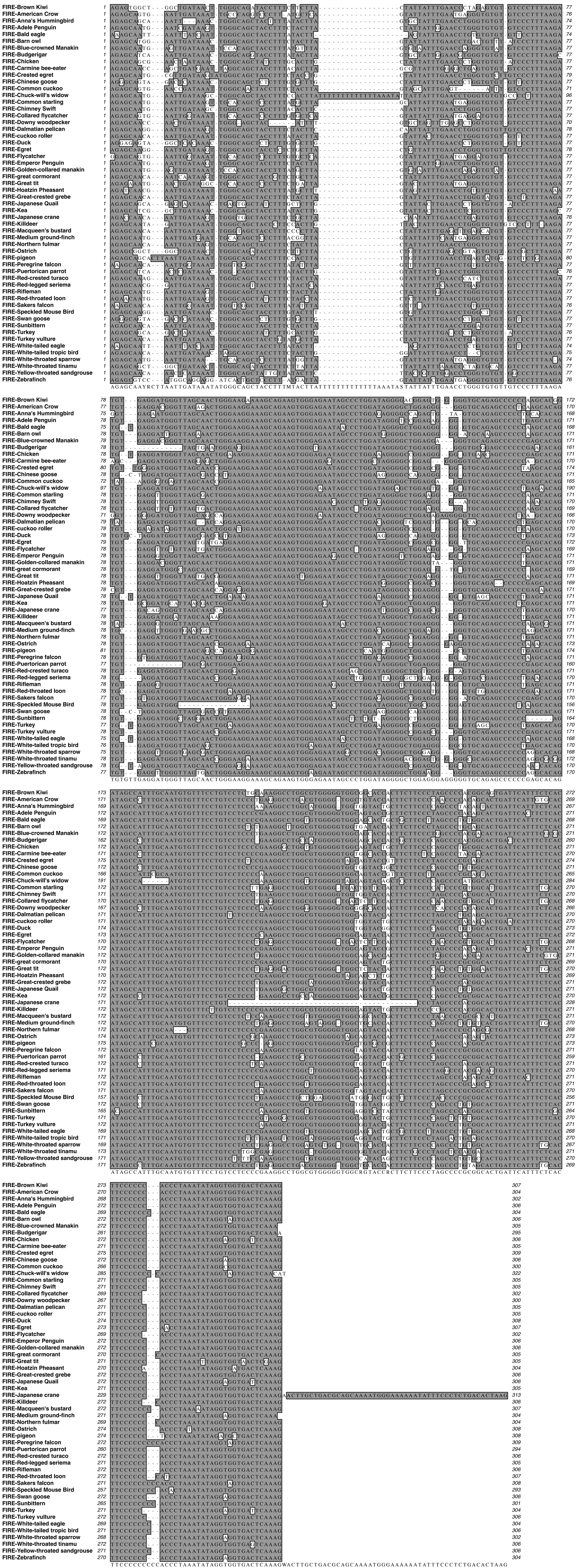

Figure S2

Formatted Alignments

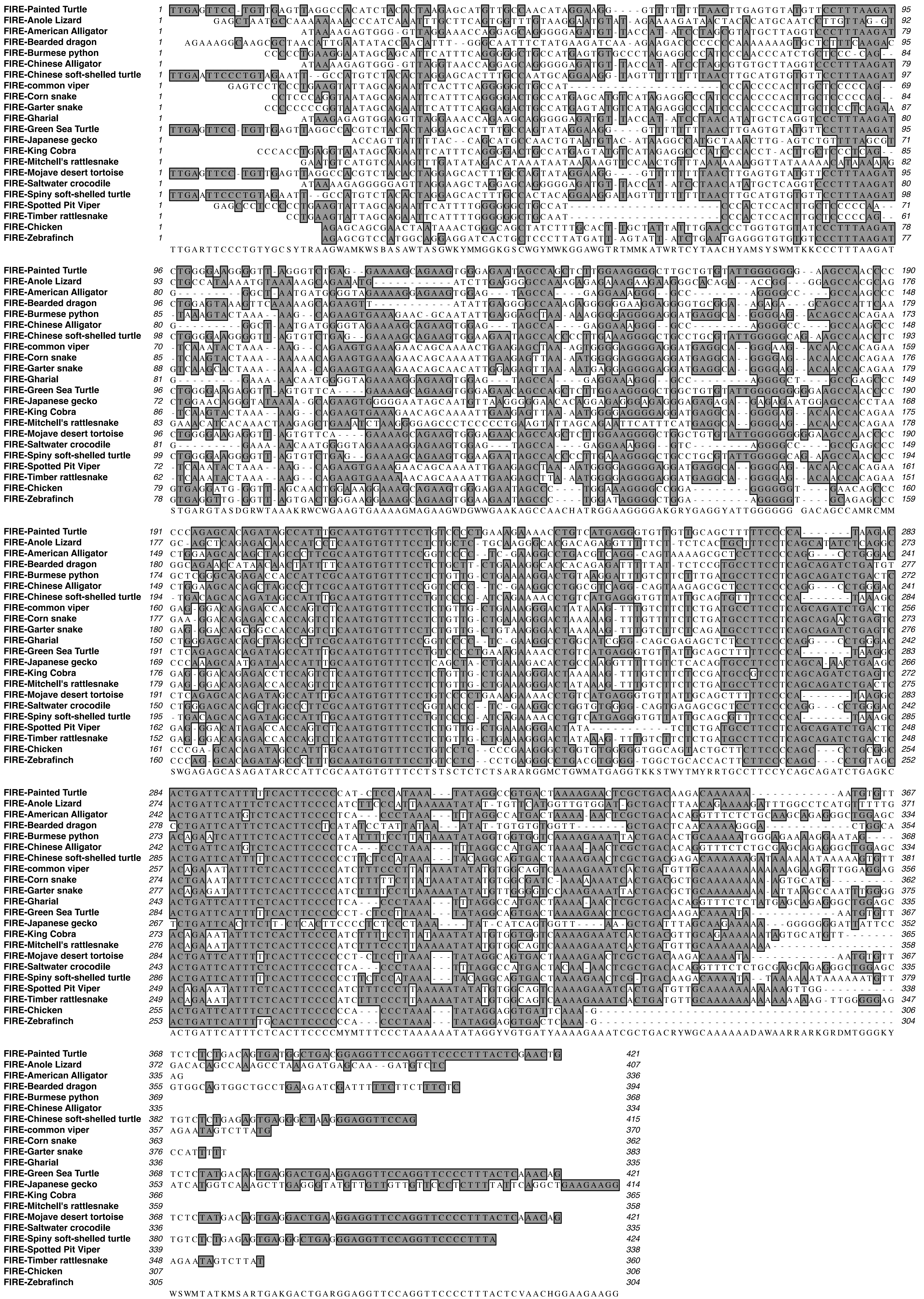

Figure S3

Formatted Alignments

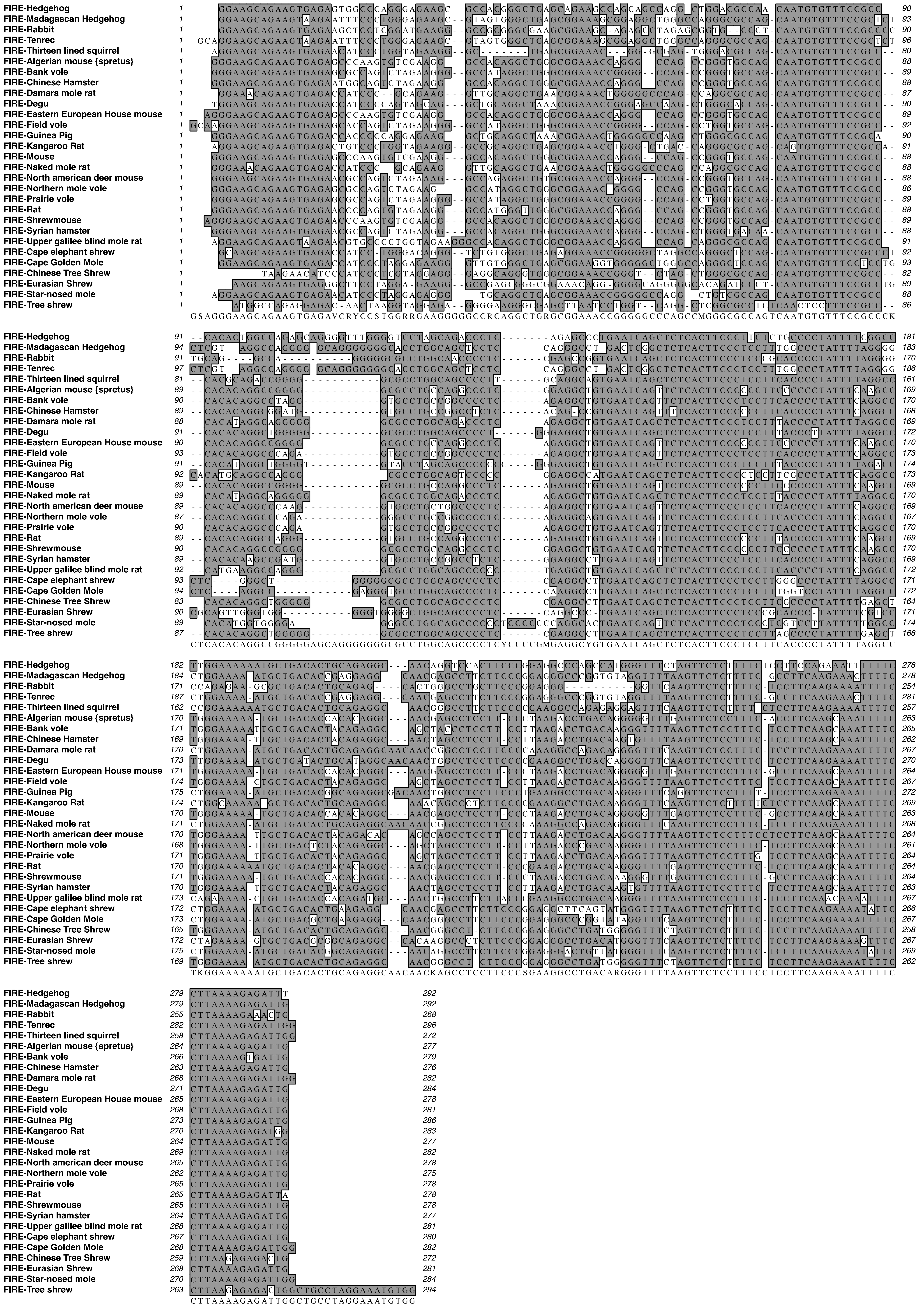

Figure S4

Formatted Alignments

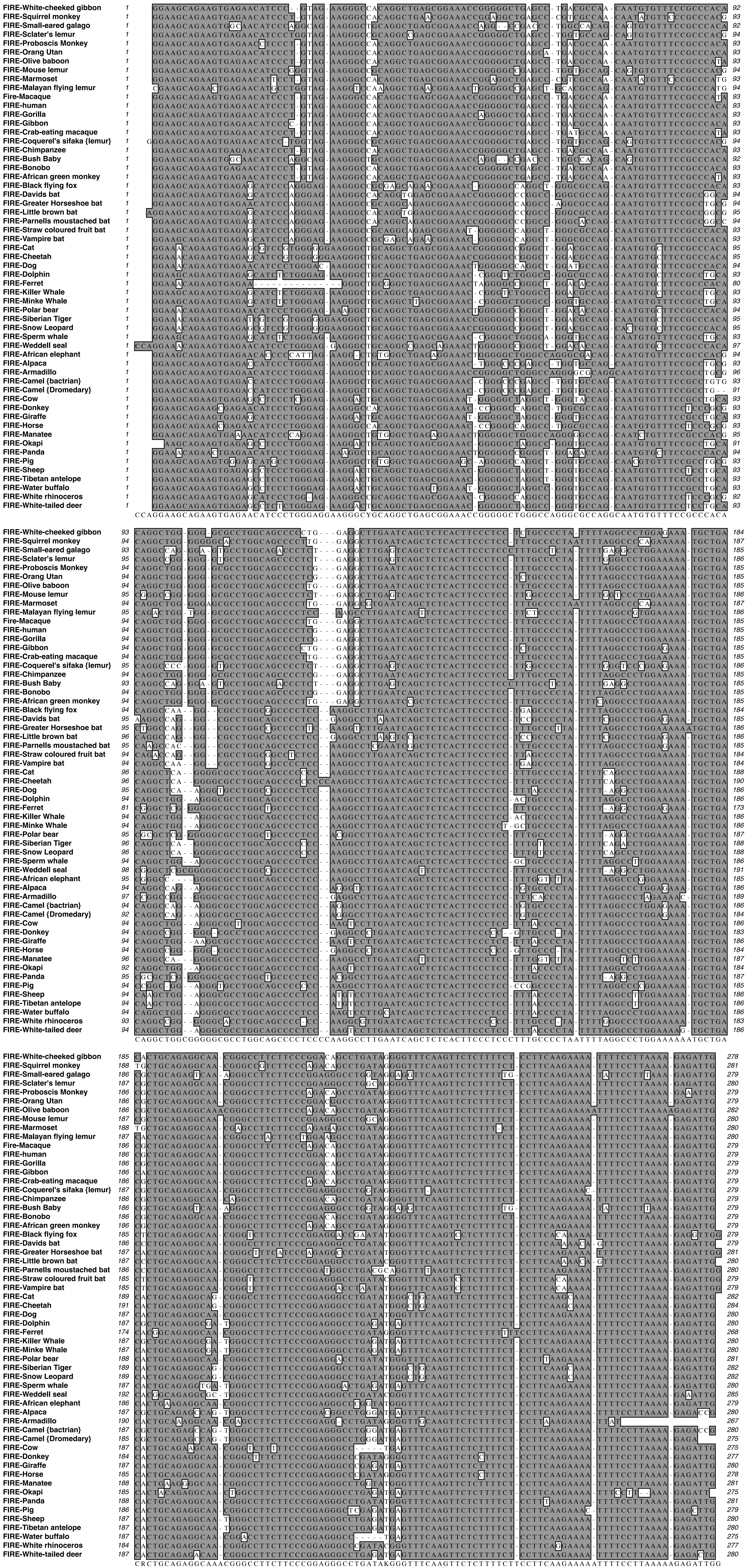

Figure S5

Formatted Alignments

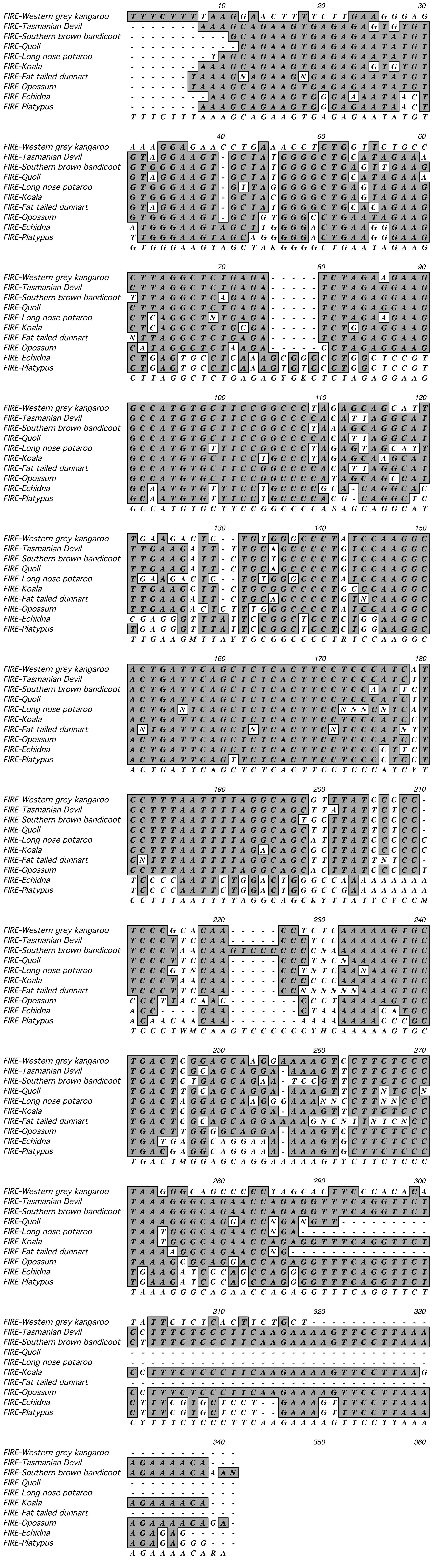

Supplement: Supplementary file 1 — Supplementary Figures and Tables [file 41598_2017_15999_MOESM1_ESM.pdf]
